# Synthesis, X-ray Crystallography, Spectroscopic Characterizations, Density Functional Theory, and Hirshfeld Surface Analyses of a Novel (Carbonato) Picket Fence Iron(III) Complex

**DOI:** 10.3390/molecules29163722

**Published:** 2024-08-06

**Authors:** Mondher Dhifet, Bouzid Gassoumi, Maxim A. Lutoshkin, Anna S. Kazachenko, Aleksandr S. Kazachenko, Omar Al-Dossary, Noureddine Issaoui, Habib Nasri

**Affiliations:** 1 Laboratory of Physical Chemistry of Materials (LR01ES19), Faculty of Sciences of Monastir, University of Monastir, Avenue of the Environment, Monastir 5019, Tunisia; mondherdhifet_2005@yahoo.fr (M.D.); habib.nasri@fsm.rnu.tn (H.N.); 2 Faculty of Sciences of Gafsa, University of Gafsa, Sidi Ahmed Zarrouk, Gafsa 2112, Tunisia; 3 Laboratory of Advanced Materials and Interfaces (LIMA), Faculty of Science of Monastir, University of Monastir, Avenue of Environnment, Monastir 5000, Tunisia; gassoumibouzid2016@gmail.com; 4 Institute of Chemistry and Chemical Technology, Krasnoyarsk Scientific Center, Siberian Branch, Russian Academy of Sciences, Akademgorodokst, 50/24, 660036 Krasnoyarsk, Russia; maximsfu@yahoo.com (M.A.L.); kaalla@list.ru (A.S.K.); 5 Institute of Non-Ferrous Metals, Siberian Federal University, pr. Svobodny, 79, 660041 Krasnoyarsk, Russia; leo_lion_leo@mail.ru; 6 Department of Physics and Astronomy, College of Science, King Saud University, P.O. Box 2455, Riyadh 11451, Saudi Arabia; omar@ksu.edu.sa; 7 Laboratory of Quantum and Statistical Physics LR18ES18, Faculty of Sciences of Monastir, Avenue of the Environment, Monastir 5079, Tunisia; 8 Higher Institute of Computer Sciences and Mathematics of Monastir, University of Monastir, Monastir 5000, Tunisia

**Keywords:** iron(III) porphyrin, XRD, FT-IR, DFT, FMO, HS

## Abstract

An Fe(III)-carbonato six-coordinate picket fence porphyrin complex with the formula [K(2,2,2-crypt)][Fe^III^(TpivPP)(CO_3_)]·C_6_H_5_Cl·3H_2_O (**I**) has been synthesized and characterized by UV-Vis and FT-IR spectra. The structure of (carbonato)(α,α,α,α-tetrakis(*o*-pivalamidophenyl)porphinato)ferrate(III) was also established by XRD. The iron atom is hexa-coordinated by the four nitrogen atoms of the pyrrol rings and the two oxygen atoms of the CO_3_^2−^ group. Complex **I**, characterized as a ferric high-spin complex (S = 5/2), presented higher Fe-Np (2.105(6) Å) and Fe-P_C_ (0.654(2) Å) distances. Both X-ray molecular structure and Hirshfeld surface analysis results show that the crystal packing of **I** is made by C-H⋯O and C-H⋯Cg weak intermolecular hydrogen interactions involving neighboring [Fe^III^(TpivPP)(CO_3_)]^−^ ion complexes. Computational studies were carried out at DFT/B3LYP-D3/LanL2DZ to investigate the HOMO and LUMO molecular frontier orbitals and the reactivity within the studied compound. The stability of compound **I** was investigated by analyzing both intra- and inter-molecular interactions using the 2D and 3DHirshfeld surface (HS) analyses. Additionally, the frontier molecular orbital (FMO) calculations and the molecular electronic potential (MEP) analyses were conducted to determine the electron localizations, electrophilic, and nucleophilic regions, as well as charge transfer (ECT) within the studied system.

## 1. Introduction

Metalloporphyrins can appear as powerful materials for the active sites of a certain number of enzymes, such as hemoproteins (hemoglobins, myoglobins, etc.) [1,2,3,4]. In the case of hemoglobin, the heme and the protein are linked together by the coordination of residues of the protein chain on the axial coordination site of iron. In general, Fe(III) can be divided into four different electronic ground states: high-spin (S = 5/2), intermediate-spin (S = 3/2), admixed-spin (S = 5/2, 3/2), and low-spin (S = 1/2). All these situations closely resemble the properties of iron (III) hemoproteins and make iron (III) porphyrins a good model for studying and understanding several properties of biomolecules. It is noteworthy that investigations on iron (III) porphyrins with bidentate axial ligands are quite rare in the literature; for example, the carbonate is not toxic to the environment. The results obtained from the structural and spectroscopic investigation of the picket fence derivative give some idea of the relative importance of the bidentate ligand vis-à-vis the stereochemical and electronic properties of the iron(III) high-spin porphyrin species. These results provide more insight into the geometry and the electronic structure of the high-spin iron(III) porphyrin derivatives with bidentate oxyanionic ligands.

Increased equatorial Fe-Np distance results in a higher spin state of the iron(III) porphyrins. This increase in distance leads to a significant decrease in the interaction between the iron d_x_^2^_−y_^2^ and porphyrin a_2u_ orbitals [5]. In general, hex-coordinated Fe(III) porphyrinates with anionic ligands can be observed in two different electronic configurations: high-spin (S = 5/2) or low-spin (S = 1/2) [6]. The five 3d orbitals of iron(III) separate into two energy groups: those of higher energy (d_x_^2^_−y_^2^ and d_z_^2^) and those of lower energy (d_xy_, d_xz_ and d_yz_). This separation highlights the electronic spin state of the iron atom in the triflate complex, but in the [K(2,2,2-crypt)][Fe^III^(TpivPP)(CO_3_)] complex, the spin is strong (S = 5/2).

Complex **I** was characterized by both spectroscopic UV/Vis and FT-IR. The molecular structure of this new hexacoordinated iron(III) metalloporphyrin was determined by single crystal X-ray diffraction (XRD). The Hirshfeld surface examination and DFT calculations of compound **I** confirm the high-spinstate. Our study focused on the picket fence porphyrin due to its ability to stabilize ionic ligands bonded to the iron atom, making it a suitable choice for our investigation [7].

## 2. Results and Discussion

### 2.1. Experimental

#### 2.1.1. Materials and Methods

All other solvents and reagents are used in specific cases, either to dissolve complexes or to facilitate obtaining the synthesized products. Depending on their uses, they must be treated and distilled in some cases, while in other cases, they are used commercially without any treatment.

UV/Vis spectral data for the complex were collected using WinASPECT PLUS (SPECORD PLUS version 4.2). The FT-IR spectra were carried out in the solid state on a Perkin-Elmer model 398 instrument, ranging from 4000 to 400 cm^−1^. The crystals obtained after the diffusion of pentane in chlorobenzene were ground with dry KBr to form pellets (1–2 mg of the sample per 100 mg of KBr).

Single crystal XRD data were collected by a Bruker-Nonius FR590 Kappa CCD diffractometer. Hirshfeld surface (HS) analysis is usually performed using Crystal Explorer 3.15 [8,9]. This program generates HS and 2D fingerprint plots of a given compound using its crystallographic information file, or CIF. The “d_norm_” Hirshfeld surface is obtained by the combination of the normalized distances from the closer atom inside the surface “d_i_” and the outside of the surface “d_e_” to the HS surface, showing all the intermolecular contacts of the crystal structure [10,11].

#### 2.1.2. Preparation of [K(2,2,2-crypt)][Fe^III^(TpivPP)(CO_3_)]·C_6_H_5_Cl·3H_2_O

Cryptand-222 (4,7,13,16,21,24-hexaoxa-l,l0-diazabicyclo[8.8.8]hexacosane (86.59 mg, 0.23 mmol) and potassium carbonate K_2_CO_3_ (55.28 mg, 0.40 mmol) were mixed together for 1 h in the chlorobenzene (V = 10 mL). [Fe^III^(TpivPP)Cl] [12] (25 mg, 0.023 mmol) in the C_6_H_5_Cl (V = 5 mL) was added to the carbonato/cryptand-222 and stirred for 3 h. The hex-coordinate (carbonato)iron(III) porphyrin complex was precipitated by the addition of dry pentane.

Elemental analysis calculated for C_89_H_107_ClN_10_O_14_FeK (molecular weight = 1671.285), C 63.96%, H 6.45%, N 8.38%; found: C 64.81%, H 6.11%, N 8.49%; UV/Vis: λ_max_ (nm, chlorobenzene, logε): 424 (5.87), 576 (4.60), 607 (4.52), FT-IR (solid, cm^−1^): 3403 (s) [ν(N-H) Pivaloyl], 2960-2871 (s), [ν(C-H) Porph.], 1680 (s) [ν(C=O) Pivaloyl], 1636 (w) [ν_1_(C-O) Carbonate], 1354 [ν_2_(C-O) Carbonate], 1104 (vs) [ν(CH_2_-O-CH_2_ Cryptand-222], 988 (s) [δ(CCH) Porph.].

#### 2.1.3. X-ray Crystallography

The crystals of the carbonato iron (III) are obtained by slow diffusion in test tubes. These crystals are washed and rinsed with pentane, then dried and well visualized on the electronic microscope while eliminating impurities. Single crystal mounted on the goniometric head using a Bruker-AXS APEX2 2014 [13]. Data were collected at room temperature using the radiation Kα of Mo (λ_Mo_ = 0.7107 Å). Complex **I** crystallizes in the monoclinic system (*P2*_1_/*n*) with four formulas per cell (Z = 4).

The X-ray diffractionstructure was resolved using the direct method SIR-2004-1.0 [14] and refined against *F*^2^ using the SHELXL-97 package [15]. During the refinement of the structure complex **I**, four disorder problems were encountered: (a) the *tert*-butyl group of one picket is disordered over two orientations C29A-C30A-C31A/C29B-C30B-C31B, with a major position occupancy of 0.512(7); (b) the one O atom of the TpivPP is occupying/disordered over two orientations O1A/O1B with a major position occupancy of 0.529(1); and (c) the two oxygen atoms of the CO_3_^2−^ group are disordered in two positions (O5A-O6A/O5B-O6B) with a major position occupancy of 0.548(1).

The anisotropic displacement ellipsoids of complex **I** were very extended, which indicates a static disorder. For fragments involving these atoms, the ISOR/SIMU restraints [16] are used, which explains the huge number of restraints. All hydrogen atoms were placed using assumed geometry, with C-H_aromatic_ = 0.95 Å and C-H_methyl_ = 0.98 Å. The displacement parameters of the H atoms were set to 1.2 (1.5 for methyl). The geometrical calculations were carried out using the program PLATON (Spek, 2002) [17]. The molecular drawings were made using ORTEP3 for Windows [18], and the packing diagrams were generated using the software MERCURY 4.0 [19].

To model the structure of compound **I**, we omitted the disordered chlorobenzene and aqua solvent molecules using the PLATON software [20]. It is obtained from our solvent-accessible voids with a volume of 4 × 229.25 Å^3^, and the number of electrons per unit cell generated by squeeze was 4 × 22 e. The corresponding results for our compound are given in Table 1.

### 2.2. Synthesis of Complex I

The synthesis of picket fence porphyrin was made according to the reported method [12]. The formula of this species is 4α-H_2_TpivPP, but it is usually written as H_2_TpivPP. Scheme S1 illustrates the different steps leading to the preparation of this *meso*-arylporphyrin, which is known to protect iron (II) porphyrins with small axial ligands that are unstable using “non-protected” porphyrin such as H_2_TPP (*meso*-tetraphenylporphyrin) or H_2_OEP (octaethylporphyrin). This is the case of the [Fe^II^(TpivPP)(l-MeIm)O_2_] [12] (1-MeIm = 1-methylimidazole) complex, where the oxo group is located inside the pocket of the TpivPPporphyrinate, leading to the stability of this species.

**Scheme 1:**
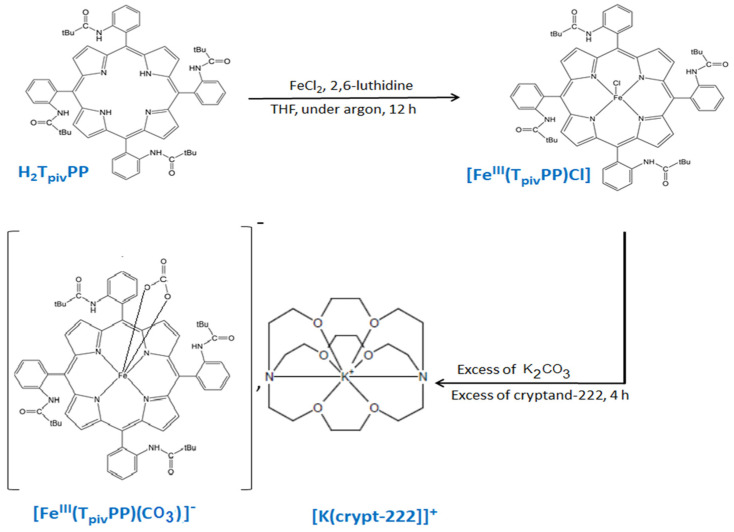
Scheme of the preparation of [Fe^III^(TpivPP)Cl] [12] and complex **I**.

The preparation of Fe(III)-chlorido with the formula [Fe^III^(TpivPP)Cl] was prepared according to the reported procedures [12]. In Scheme 1, the synthetic procedure of [K(2,2,2-crypt)][Fe^III^(TpivPP)(CO_3_)]·C_6_H_5_Cl·3H_2_O, namely the (carbonato) [α,α,α,α-tetrakis(*o*-pivalamidophenyl)porphyrinato]iron(III) chlorobenzene trihydrate, is shown. Furthermore, in the formula of **I**, we omitted the disordered chlorobenzene and aqua solvent molecules using the squeeze procedure of PLATON [20], so that the formula is [K(2,2,2-crypt)][Fe^III^(TpivPP)(CO_3_)] (**I**).

**Scheme 2:**
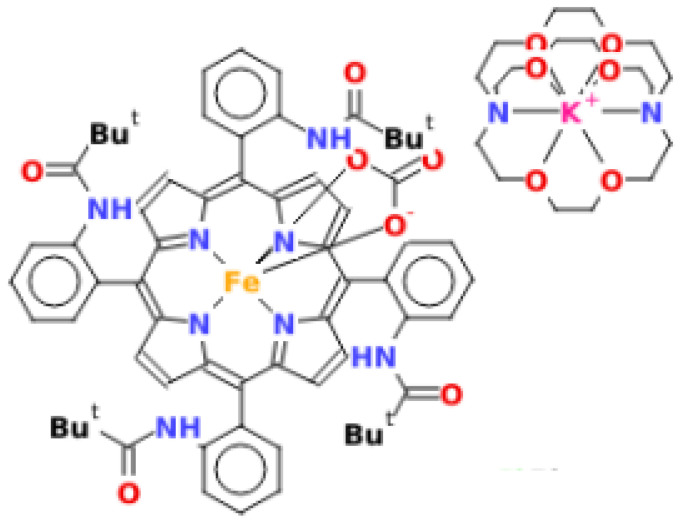
Two-dimensional chemical graph of complex **I**.

In Scheme 2, the chemical graph of complex **I** is shown.

### 2.3. Spectroscopic Characterizations

**Figure 1:**
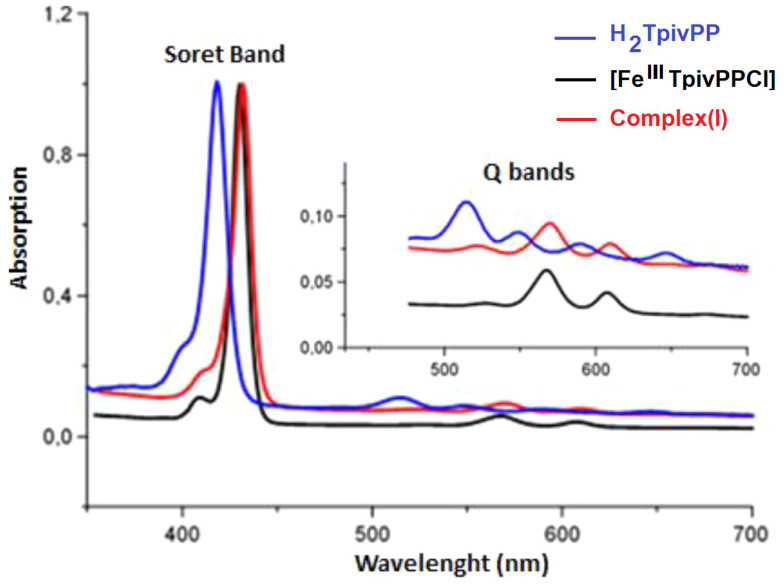
Electronic spectra of H_2_TpivPP, [Fe(TpivPP)Cl], and complex **I** in C_6_H_5_Cl solution.

The UV-visible spectra of H_2_TpivlPP, [Fe^III^(TpivPP)Cl], and complex **I** are illustrated in Figure 1. Table S1 compares the λ_max_ values with those of other *meso*-porphyrin-iron metalloporphyrin compounds.

The concentration used for the spectrum in the Soret band region is 1.5 × 10^−6^ M, while the concentration used in the Q band area is 6.1 × 10^−5^ M. This spectrum presents a Soret band around 424 nm and a β and α bands around 576 nm and 607 nm, respectively.

These results are similar for iron(III) *meso*-porphyrin complexes with anionic axial ligands [21].

The optical gap (E_g-opt_) value of complex **I** was determined using the tangent method according to the formula:

E_g-opt_ = 1239.8/λ_Lim_

where λ_Lim_ is the intersection between the tangent to the highest Q band and the horizontal (Figure S1). This experimental value is 1.907 eV, which is common for porphyrins and metalloporphyrins and indicates the semi-conductor character of our carbonato iron(III) complex.

**Figure 2:**
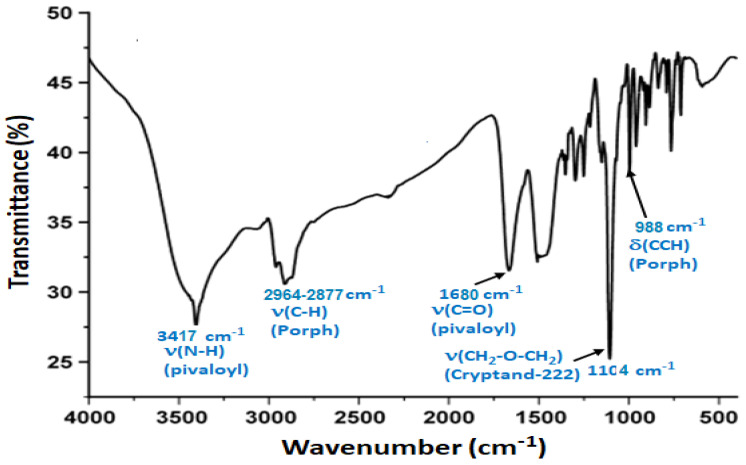
Experimental IR spectrum of complex **I**.

Complex **I** shows an intensive absorption peak in the infrared spectrum located at 1104 cm^−1^, which corresponds to the -CH_2_-O-CH_2_- group of [K(crypt-222)]^+^ (see Figure 2). It exhibits typical absorption bands of H_2_TpivPP with the ν(C=O) of the carbonyl group of the pivaloyl and δ(CCH) bending frequencies at 1680 cm^−1^ and 988 cm^−1^, respectively. In the same IR spectrum of complex **I**, two weak bands at 1636 and 1356 cm^−1^ attributed to the ν_1_(C-O) and ν_2_(C-O) vibrations confirm the existence of the (CO_3_)^2−^ ligand.

### 2.4. Structural Properties of [K(2,2,2-crypt)][Fe^III^(TpivPP)(CO_3_)] (***I***)

**Figure 3:**
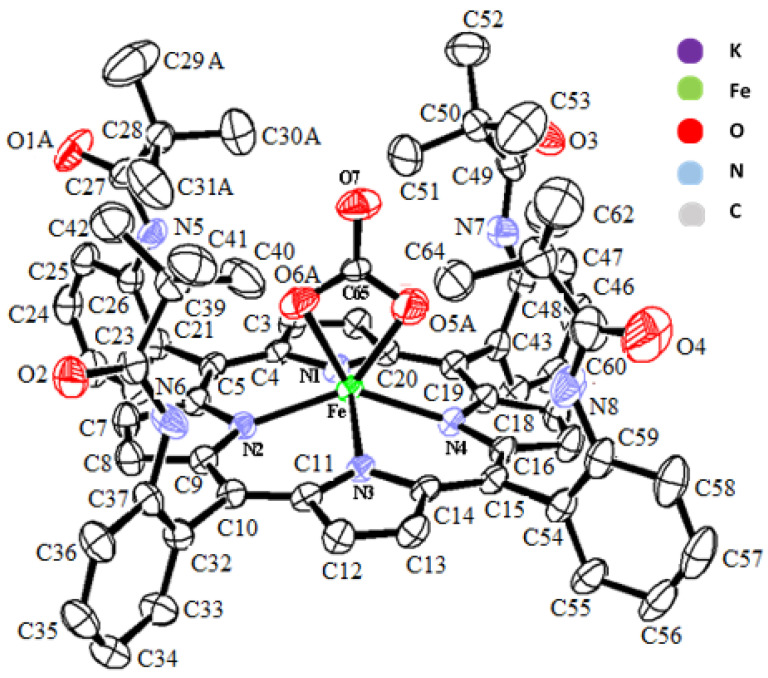
Ortep diagram showing the [Fe^III^(TpivPP)(CO_3_)]^−^ ion complex. Thermal ellipsoids are drawn at the 30% probability level. Only the major positions of the disordered fragments are shown, and the hydrogen atoms have been omitted for clarity.

The asymmetric unit of (**I**) contains the anioncomplex [Fe^III^(TpivPP)(CO_3_)]^−^ with one [K(2,2,2-crypt)]^+^ cation, one C_6_H_5_Cl solvent, and three molecules of water. A disordered chlorobenzene and three molecules of water, which could not be modeled, were removed from the lattice of **I** by using the squeeze procedure [20]. The ortep diagram shows the structure of the anioncomplex (see Figure 3). The geometric parameters (bond length (Å) and angles (°)) of complex **I** are reported in Table 2.

The [K(crypt-222)]^+^ counterion is shown in Figure S2. The K-O (crypt-222) and K-N (crypt-222) distances [2.818 (6) Å and 3.039 (6) Å, respectively] are in agreement with the literature values [21].

The geometric parameters Fe-Np, Fe-X_L_, and Fe-P_C_ in compound **I** and a selection of some other six-coordinate iron(III) high-spin (S = 5/2) and low-spin (S = 1/2) porphyrin complexes are given in Table 3.

**Figure 4:**
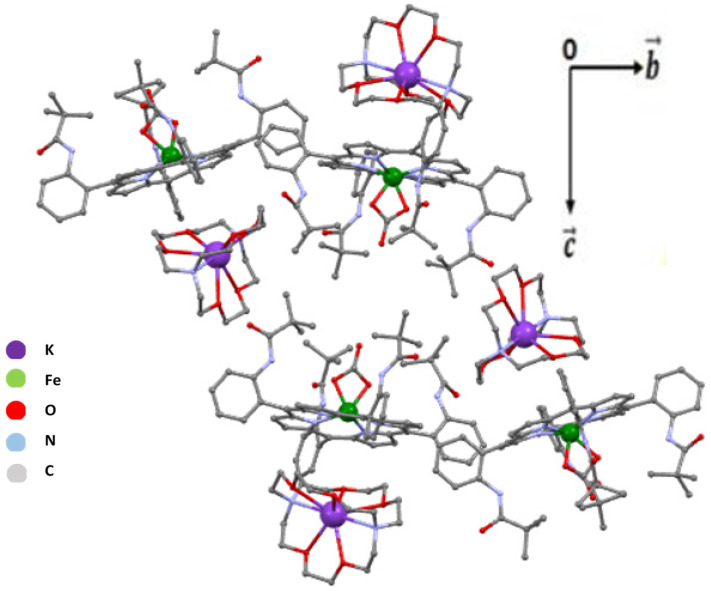
Projection of the packing diagram of complex **I** down the a axis. Only the major positions of the disordered fragments are shown, and the hydrogen atoms have been omitted for clarity.

In complex **I**, Fe-Np (2.105(6) Å) and Fe-P_C_ (0.654(2) Å) distances are in the normal range of 2.048–2.125 Å and 0.20–0.75 Å, respectively. This value confirms clearly that compound **I** is a hexacoordinated metalloporphyrin with highspin (S = 5/2). However, six-coordinate iron (III) low-spin porphyrin species present lower Fe-P_C_ and Fe-Np distances. Figure 4 illustrates the crystal packing of complex **I** perpendicular to the a axis. 

**Figure 5:**
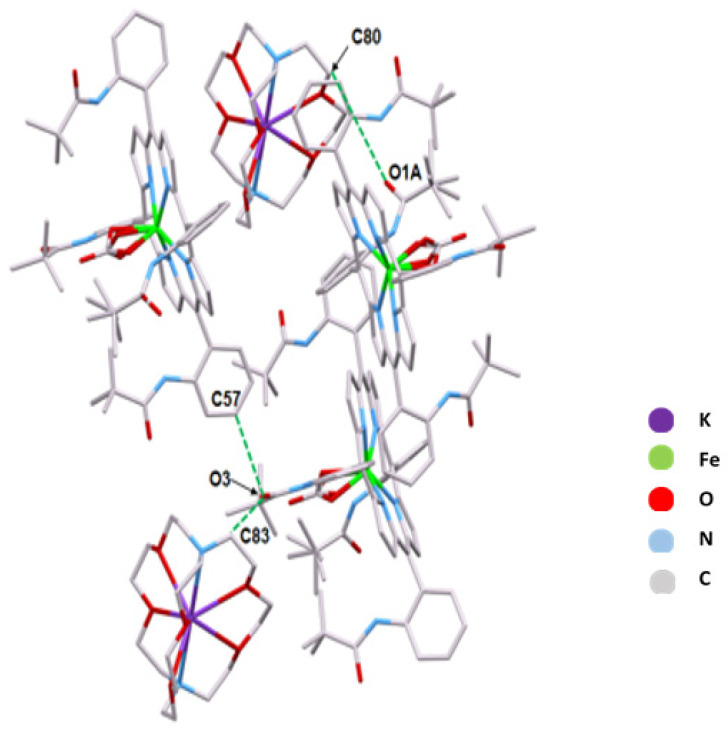
Drawing illustrating the C–H⋯O intermolecular interactions in complex **I**.

**Figure 6:**
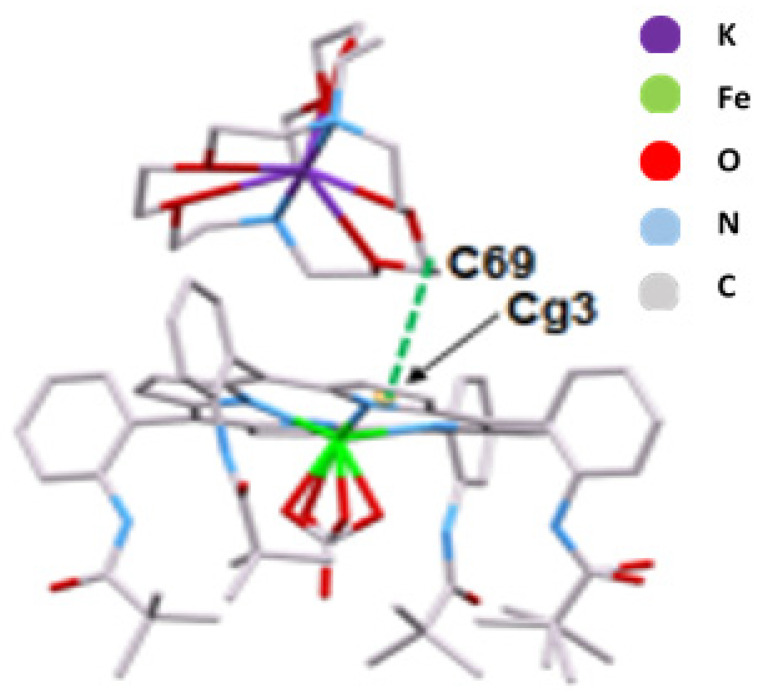
Drawing illustrating the C69–H69A⋯Cg3 intermolecular interactions in complex **I**.

In order to investigate the intermolecular interactions within the crystal packing of complex **I**, we used the PLATON program [17], as described just above, to obtain a good visualization of these intermolecular interactions (see Figure 5 and Figure 6).

**Figure 7:**
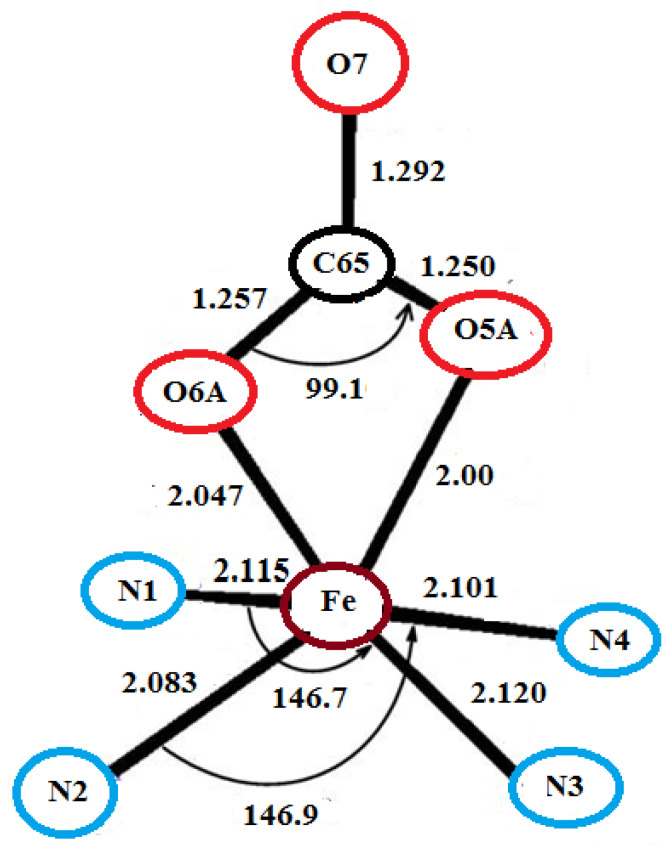
Geometric parameters (distances (Å)/angles (°)) of the iron coordination polyhedron.

Figure 7 represents the coordination polyhedral and the environment of iron. The distances between the two O atoms O5A/O6A of the CO_3_^2−^ and the iron atom are 2.047(15) Å and 2.00(2) Å, respectively. The two values of bond length are almost equal and are slightly shorter than the third distance Fe-O7 (CO_3_^2−^) bond length. The long bond length observed in our case is probably due to the type and environment of the porphyrin used.

**Figure 8:**
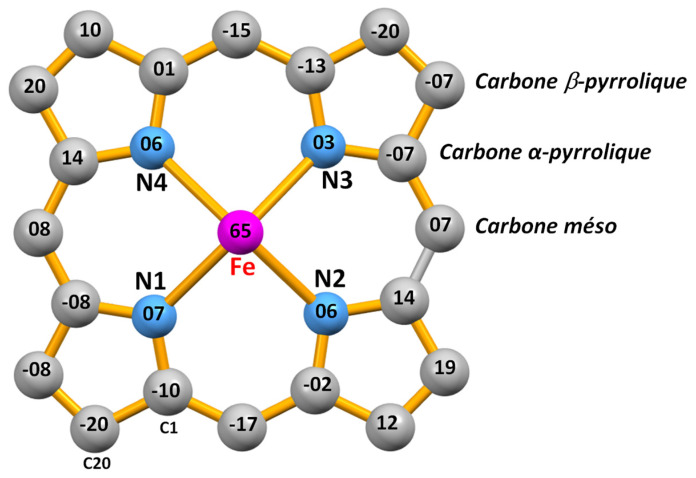
Porphyrin core in the [Fe^III^(TpivPP)(CO_3_)]^−^ illustrating the displacements of each atomin units of 0.01 Å.

The porphyrin core is shown in Figure 8. The Fe-porphyrin and the number of coordinations for iron have several properties for the irregular and non-planar conformation of the porphyrin core. The diagram highlights significant saddling and moderate doming distortions of the macrocycle [27].

## 3. DFT-D3 Investigations of Compound I

### 3.1. Computational Details

The DFT calculations were made based on the crystallographic CIF (Crystallographic Information File) using the DFT/B3LYP-D3/LanL2DZ [28,29,30] level of theory, as implemented in Gaussian 09 [31]. The represented figures have been selected using Gauss View 5 [32]. Analysis of HOMO and LUMO orbital occupancies gives insights into the electron localization on the surface of the material and the charge transfer mechanisms within the complex [33]. The molecular electrostatic potential (MEP) was determined to investigate the electrophilic and nucleophilic sites on the compound’s surface. The Hirshfeld surface (HS) analyses were deeply studied to elucidate the responsible inter- and intra-molecular interactions that contribute to the stability of the compound.

### 3.2. Optimized Structure of [K(crypt-222)][Fe^III^(TpivPP)(CO_3_)] (***I***)

**Figure 9:**
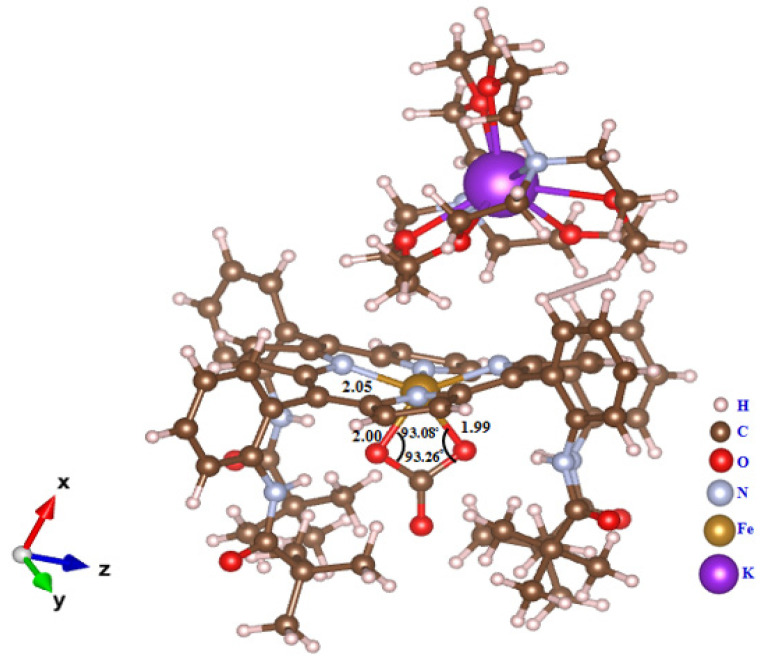
Optimized structure of studied compound **I**.

Figure 9 depicts a side view of our studied compound. Table 4 presents a compilation of selected experimental and calculated bond lengths and angles within the coordination sphere polyhedral surrounding the iron(III) central ion of complex **I**. The comparison between experimental and theoretical values for this dihaptocarbonato iron(III) complex reveals a high degree of proximity. Specifically, the Fe-Np distances are measured at 2.105 (6) Å experimentally and 2.052 Å theoretically, while the Fe-O(axial ligand) distances are recorded at 2.00(2)/2.047(2) Å experimentally and 1.992/2.001 Å theoretically. This difference between the experimental and theoretical distance values may be due to the fact that the calculated values are obtained assuming a gas phase, while the experimental values were obtained from the X-ray crystal structure (solid state).

Additionally, the Fe-O5A-C65 angle is determined to be approximately 93.26°, closely aligning with the experimental value of 95.50°. It is observed that the H⋯H interaction is low bonding, formed between the [K(crypt-222)] and the compound. These findings support the notion of strong agreement between theoretical predictions and experimental observations, underscoring the robust stability and effective group interactions within complex **I**.

### 3.3. HOMO/LUMO and Global Reactivity Investigations

**Figure 10:**
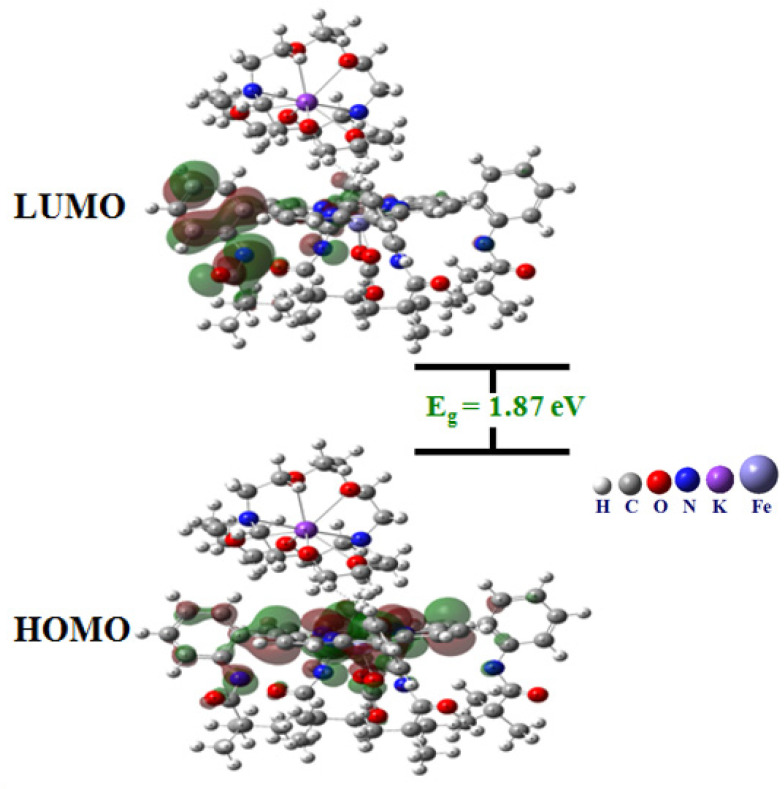
HOMO/LUMO orbital of the current compound.

The HOMO and LUMO iso-surfaces are key parameters for determining electron acceptance or donation ability in the compound [34,35,36,37]. Molecular orbitals (FMO) provide a clear idea of the electron occupations in the studied material and electronic charge transfer (ECT), aiding in the understanding of the electronic properties of materials [38]. Furthermore, the gap energy gives a clear idea concerning the chemical reactivity, kinetic stability, global hardness-softness characteristics, and optical polarizability of the compound. Moreover, the FMO analysis offers insights into the acceptor regions, which is beneficial for biological applications. The HOMO and LUMO orbitals are shown in Figure 10. Analysis of the HOMO orbital reveals an electron accumulation around the central metal Fe and the anionic axial ligand. Subsequently, these lone pairs of electrons cross the forbidden band to the LUMO orbitals, where they are accumulating along the ligand. This discovery suggests a significant charge transfer occurring between the Fering and the neighboring ligand. The localization of high electron populations on the surface is necessary for biological mechanisms. The ε_HOMO_ε_LUMO_ energies were found to be −5.82 eV and −2.50 eV, respectively. The gap energy was found to be 1.87 eV. This indicates the chemical and kinetic stability of our material, as well as its polarizability properties. Additionally, global reactivity parameters were calculated using Koopman’s theorem, with the results presented in Table 5.

A low global hardness value of approximately 0.93 eV was obtained, and it can be concluded that electrons can easily cross from S0 (ground state) to S1 (excited state). The chemical potential (µ) and hardness (η) values are around −3.90 eV and 0.93 eV, as shown in Table 5, suggesting the stability of the compound, a result supported by molecular electrostatic potential (MEP) and FMO analysis. The significant electronegativity (χ = 3.90 eV) indicates its potential for electronic charge transfer with the target receptor. Furthermore, the high electrophilicity index (ω) of 8.17 eV suggests a highly inhibitory system.

### 3.4. Molecular Electrostatic Potential (MEP) Analysis

The molecular electrostatic potential (MEP) is a technique that is suitable for determining and understanding the electrophilic (negative potential) and nucleophilic (positive potential) sites that characterize our compound. MEP is an important tool for identifying acceptor and donor attack sites that are useful for sensor and biological applications.

**Figure 11:**
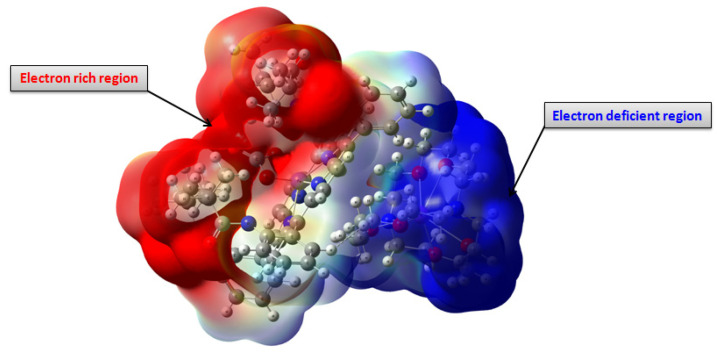
MEP plot of studied compound **I**.

The MEP is represented by color codes identifying the sign of potential in each region, ranging from red to blue. The 3D MEP plot is shown in Figure 11. It can be seen that a dark red color (indicating positive electrostatic potential) surrounds the dihaptocarbonato near the central metal Fe, suggesting a high electronic localization in this area. Additionally, the clear presence of a positive electrostatic potential (blue region) around the iron and neighboring groups indicates the electrophilic nature of this area. This finding concluded that a highly electronic/energy charge transfer takes place on the surface of the studied compound, as confirmed by FMO analyses. The identification of the existence of double electrophilic and nucleophilic sites resulted in the exceptional ability of [K(2,2,2-crypt)][Fe^III^(TpivPP)(CO_3_)] to function as both electron donors and acceptors. This highlights the high capacity of this material for potential applications in sensors and biological applications.

### 3.5. Hirshfeld Surface (HS) Analysis

Hirshfeld surface (HS) and two-dimensional (2D) fingerprint plots are robust analytical tools that provide a comprehensive insight into intra- and intermolecular interactions within the crystal lattice [39,40]. The HS serves as a valuable resource for comprehending the co-existing forces between constituent groups within the crystal structure, which ultimately dictate the stability of the molecular complex [41]. The 2Dfingerprint plot is generated as a d_e_ in the function of d_i_, representing the percentage of atomic contacts. The HS iso-surfaces offer a visual representation of interaction through a color-coded scheme, with red, white, and blue spots denoting varying degrees of interaction intensity. Dark red regions signify strong hydrogen bonding interactions; white regions indicate proximity in van der Waals contacts; and blue regions represent longer-range interactions. The d_norm_is located between −0.4481 Å and −0.8821 Å, the shape index is −1 to 1 Å, and the curvedness varies from −4 to 0.4 Å. The normalized distance d_norm_ is calculated using the following formula [42,43]:

$$d_{norm}=\frac{d_{i}-r_{i}^{vdw}}{r_{i}^{vdw}}+\frac{d_{e}-r_{e}^{vdw}}{r_{e}^{vdw}}$$

**Figure 12:**
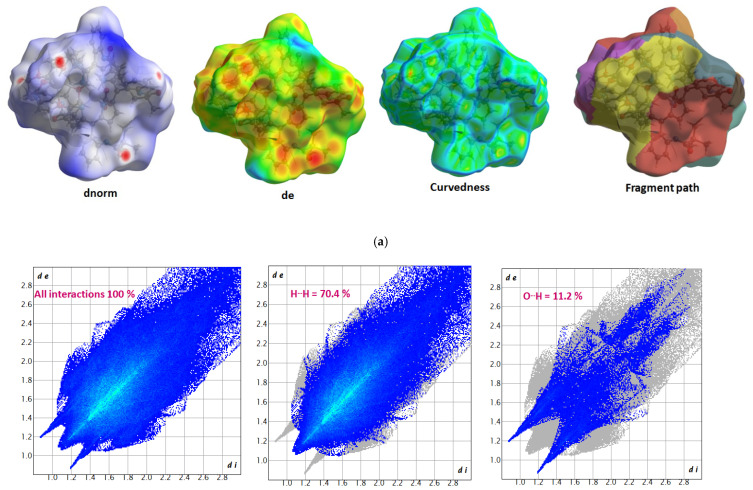

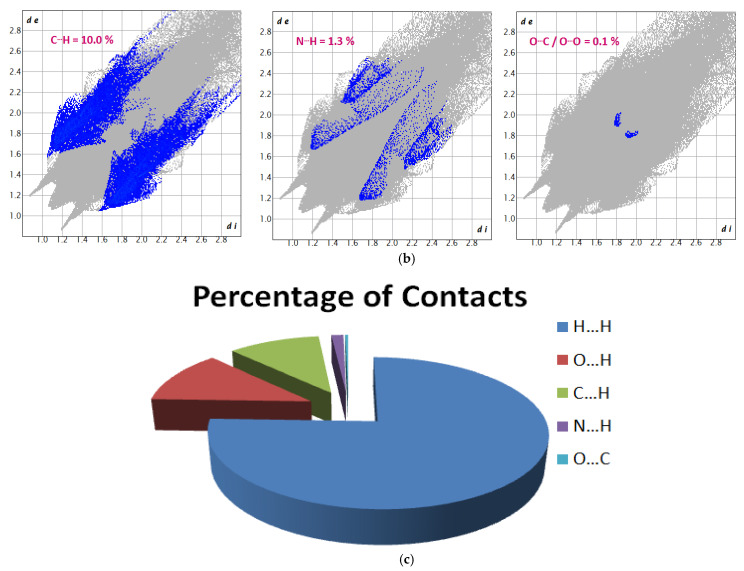
(**a**) d_norm_, d_e_, shape index, and curvedness; (**b**) the 2Dfingerprint plot; and (**c**) the 2Ddiagram of the percentage of contacts in the current complex **I**.

The d_norm_ is intricately linked to both d_e_ and d_i_, where the parameters $r_{i}^{\mathrm{vdw}}$ and $r_{e}^{\mathrm{vdw}}$ delineate the van der Waals radii of the atom. The d_norm_, d_e_, curvedness, fragment path, and 2Dfingerprint plot have been illustrated in Figure 12a,b. The percentages of contacts have been graphically represented in a 2D diagram, as depicted in Figure 12c. H⋯H interactions dominate the Hirshfeld surface, accounting for 77.4% of the total surface area. The next significant contributions are C⋯H and O⋯H interactions in the crystal packing, making up 10.0% and 11.2%, respectively, as depicted in the 2Dfingerprint plot. This finding suggests that these interactions may be enhanced by the electron delocalization on [K(2,2,2-crypt)][Fe^III^(TpivPP)(CO_3_)] surfaces. Interatomic contacts involving N⋯H, O⋯C, and O⋯O exhibit lower contributions in the crystal structure, with percentages of 1.3% and 0.1%, respectively. Analysis of the HS and 2Dfingerprint plots revealed that the robust stability of the functional groups within our compound may provide excellent electronic properties for our novel system.

## 4. Conclusions

Complex **I** was synthesized and characterized by UV-vis and FT-IR spectroscopic methods. The high-spin of complex **I** is confirmed by the equatorial mean distance of 2.105(6) Å between Fe(III) and the nitrogen atoms of the pyrrole rings. These studies validate that six-coordinated high-spin iron(III) porphyrinates, such as [Fe(Porph)(X)]^−^(X corresponds to the bidentate anionic ligand), exhibit this characteristic. Additionally, the N atoms of the four pyrrole rings, the α-carbon positions of these pyrroles, and the O atoms of the carbonate (CO_3_^2−^) axial ligand contribute to the overall reactivity of this high-spin (S = 5/2) ferric metalloporphyrin, indicating its highly reactive and electron-accepting nature. Molecular electrostatic potential (MEP) calculations provide insight into the electrophilic/nucleophilic properties of complex **I**, while frontier molecular orbital (FMO) analyses reveal significant electronic charge transfer around the central Fe(III) ion. The MEP analysis further confirms the significant electron-accepting behavior of this novel ferric porphyrinic compound. The HS analyses confirm the atomic reorganization and stability of our compound through hydrogen bonding interactions with its neighboring unit cell in the crystal packing.

## Figures and Tables

**Table 1:** Crystallographic data for complex **I**.

Empirical Formula	C_83_H_100_N_10_O_13_KFe
Formula weight, M	1649.2
T (K)	293 (2)
Wavelength(Å)	0.71073
Crystal system	monoclinic
Space group	
P2_1_/n	
Lattice constants	
*a* (Å); *b* (Å); *c* (Å)	17.932 (2); 21.484 (2); 22.924 (2)
β (°)	100.813 (3)
Volume *V* (Å^3^)	8674.8 (14)
*Z*	4
D_cal_, g/cm^3^	1.263
Absorption coefficient, *μ* (mm^−1^)	0.319
F(000)	3484
Crystal size (mm^3^)	0.30 × 0.15 × 0.10
*θ*-range for data collection	1.6–19.8
Limiting indices	−17 ≤ h ≤ 17, −20 ≤ k ≤ 20, −21 ≤ l ≤ 21
Reflexions collected/unique	82,787/7805
R(int)/R(σ)	0.1122/0.0478
Data/Restraints/Parameters	7805/17/1028
X (Flack parameter)	0.18(2)
Final *R* indices [*I* > *2σ(I)*]	^a^ *R*_1_= 0.083, *wR*_2_= 0.200
*R* indices (all data)	*R*_1_ = 0.103, w*R*_2_ = 0.213
Goodness-of-fit on *F_o_*^2^	1.047
Highest peak and deepest hole (eÅ^−3^)	1.905 and −0.787
CCDC	2,367,771

^a^ *R*_1_ = ∑||*F*_o_| − |*F*_c_||/∑|*F*_o_|.

**Table 2:** Geometric parameters (distances (Å) and angles (°)) in compound **I**.

**Iron Coordination Polyhedron**			
Fe-N1	2.115 (6)	N1-Fe-N2	85.9 (2)
Fe-N2	2.083 (5)	N1-Fe-N3	146.7 (2)
Fe-N3	2.120 (6)	N1-Fe-N4	85.0 (2)
Fe-N4	2.101 (6)	N2-Fe-N3	85.4 (2)
Fe-N_p_	2.105 (6)	N2-Fe-N4	146.9 (2)
Fe-O5A	2.00 (2)	N1-Fe-O5A	110.0 (6)
Fe-O6A	2.047 (2)	N2-Fe-O5A	136.4 (6)
N3-Fe-O5A	98.3 (6)	N3-Fe-O6A	133.8 (6)
N4-Fe-O5A	76.4 (5)	N4-Fe-O6A	118.4 (7)
N1-Fe-O6A	78.3 (6)	N2-Fe-O6A	90.6 (6)
**Carbonato ligand**			
C65-O5A	1.250 (2)	Fe-O6A-C65	91.9 (2)
C65-O6A	1.257 (2)	O5A-C65-O6A	99.1 (2)
C65-O7	1.292 (1)	O5A-C65-O7	132.8 (2)
Fe-O5A-C65	95.5 (1)	O6A-C65-O7	118.6 (2)
**Potassium-cryptand-222**			
K-N9	3.023 (7)	N9-K-N10	179.48 (2)
K-N10	3.055 (6)	N9-K-O8	59.94 (2)
K-O8	2.807 (6)	N9-K-O9	119.91 (2)
K-O9	2.839 (5)	O8-K-O9	60.15 (2)
K-O10	2.842 (2)	O8-K-O10	128.13 (2)
K-O11	2.823 (5)	O8-K-O11	100.93 (2)
K-O12	2.834 (6)	O9-K-O12	121.18 (2)
K-O13	2.767 (6)	O11-K-O12	96.55 (2)

**Table 3:** Selected structural features of several hex-coordinate (porphyrinato) iron(III) derivatives.

Complex	Fe–N_p_^a^	Fe–X_L_^b^	Fe–P_C_^c^	Ref.
*High-spin (S = 5/2) iron(III) hexacoordinated metalloporphyrins*				
[Fe^III^(TpivPP)(CO_3_)]^−^	2.105	2.047	0.654(2)	T.w.
		2.000		
[Fe^III^(TpivPP)(*η*^2^-O_2_CO)]^−^	2.125	2.005	0.749(7)	[22]
		2.031		
[Fe^III^(TPP)(*η*^2^-O_2_NO)] ^d^	2.073	2.323	0.600(1)	[23]
		2.019		
[Fe^III^(TPP)(γ^2^-O_2_NO)]	2.085	2.124	0.620(1)	[23]
		2.268		
[Fe^III^(TTP)(OTeF_5_)(THF)] ^e^	2.053	2.334	0.200	[24]
		1.967		
*Low-spin (S = 1/2) iron(III) hexacoordinated metalloporphyrins*				
[Fe^III^(TpivPP)(NO_2_)_2_]^−^	1.992	1.985	0.020	[25]
		1.985		
[Fe^III^(TpivPP)(SC_6_HF_4_)(NO_2_)]^−^	1.980	2.277	0.050(1)	[25]
		1.990		
[Fe^III^(TPP)(SC_6_HF_4_)_2_]^−^	1.998	2.312	0 ^f^	[26]
		2.312		

^a^ Fe-Np: EquatorialFe-N_pyrrole_ distance. ^b^ Fe-X_L_: Fe-axial ligand distance. ^c^ Fe-Pc: Fe-median plan of core distance. ^d^ TPP: Tetraphenylporphyrin. ^e^ OTeF_5_: Teflate. ^f^ Value of 0 required by symmetry. T.w.: This work.

**Table 4:** Selected bond lengths (Å) and bond angles (°) of [Fe^III^(TpivPP(CO_3_)]^−^ (**I**).

Iron Coordination Polyhedron		
	Experimental Values	Theoretic Values
Fe-N1	2.115(6)	2.263
Fe-N2	2.083(5)	2.021
Fe-N3	2.120(6)	2.061
Fe-N4	2.101(6)	2.052
Fe-O5A	2.00(2)	1.992
Fe-O6A	2.047(2)	2.001
C65-O5A	1.250(2)	1.367
C65-O6A	1.257(2)	1.365
C65-O7	1.292(1)	1.244
N1-Fe-O5A	110.0(6)	143.44
N1-Fe-O6A	78.3(6)	79.30
N2-Fe-O5A	136.4(6)	143.43
N2-Fe-O6A	90.6(6)	89.90
N3-Fe-O5A	98.3(6)	104.44
N3-Fe-O6A	133.8(6)	142.59
N4-Fe-O5A	76.4(5)	87.67
N4-Fe-O6A	118.4(7)	101.87
N1-Fe-N2	85.9(2)	87.16
N1-Fe-N3	146.7(2)	137.55
N1-Fe-N4	85.0(2)	88.04
N2-Fe-N3	85.4(2)	86.88
N2-Fe-N4	146.9(2)	165.89
Fe-O5A-C65	95.5(1)	93.26
Fe-O6A-C65	91.9(2)	93.08
O5A-C65-O6A	99.1(2)	107.38
O5A-C65-O7	132.8(2)	126.28
O6A-C65-O7	118.6(2)	126.32

**Table 5:** Calculated Quantum chemical parameters for the studied complex.

Quantum Chemical Parameters (eV)	
ε_HOMO_	−4.84
ε_LUMO_	−2.97
$E_{g}=\varepsilon_{LUMO}-\varepsilon_{HOMO}$	1.87
$IP=-\varepsilon_{HOMO}$	4.84
$EA=-\varepsilon_{LUMO}$	2.97
Chemical potential,	−3.90
Mulliken electronegativity, χ = −μ	3.90
Global hardness, $\eta =\frac{IP-EA}{2}$	0.93
Electrophilicity index, ω = $\frac{\mu^{2}}{2\eta }$	8.17

## Data Availability

CCDC 2367771 contains the supplementary crystallographic data for this paper. These data can be obtained free of charge via https://www.ccdc.cam.ac.uk/structures/search?Ccdc=2367771&Author=dhifet&Access=referee (accessed on 16 July 2024), or from the Cambridge Crystallographic Data Center, 12 Union Road, Cambridge CB2 1EZ, UK; fax: (+44)-1223-336-033; or e-mail: deposit@ccdc.cam.ac.uk.

## Supplementary Materials

The following supporting information can be downloaded at: https://www.mdpi.com/article/10.3390/molecules29163722/s1, The synthesis of picket fence porphyrin (H_2_TpivPP), UV/Vis spectroscopy, and X-ray molecular structure of the [K(2,2,2-crypt)]^+^ counter-ion are reported in Scheme S1 and Figures S1 and S2. Table S1. Electronic absorption data for complex I and a selection of meso-arylporphyrin compounds. Refs. [44,45,46,47,48,49,50,51,52] are cited in Supplementary Materials.
