# Supplementary material for: Synthesis, X-ray Crystallography, Spectroscopic Characterizations, Density Functional Theory, and Hirshfeld Surface Analyses of a Novel (Carbonato) Picket Fence Iron(III) Complex
Source: Molecules. 2024 Aug 6;29(16):3722. doi: 10.3390/molecules29163722 (PMC11357499; doi:10.3390/molecules29163722)

checkCIF/PLATON report

Structure factors have been supplied for datablock(s) I

THIS REPORT IS FOR GUIDANCE ONLY. IF USED AS PART OF A REVIEW PROCEDURE FOR PUBLICATION, IT SHOULD NOT REPLACE THE EXPERTISE OF AN EXPERIENCED CRYSTALLOGRAPHIC REFEREE.

No syntax errors found.   CIF dictionary   Interpreting this report

Datablock: I

Bond precision:	C-C = 0.0133 A		Wavelength=0.71073
Cell:	a=17.9320(17)	b=21.484(2)	c=22.924(2)
	alpha=90	beta=100.813(3)	gamma=90
Temperature:	293 K		

	Calculated	Reported
Volume	8674.7(14)	8674.8(14)
Space group	P 21/n	P 21/n
Hall group	-P 2yn	-P 2yn
Moiety formula	C65 H60 Fe N8 O7, C18 H36 K N2 O6 [+ solvent]	?
Sum formula	C83 H96 Fe K N10 O13 [+ solvent]	C89 H101 Cl Fe K N10 O13
Mr	1536.65	1649.19
Dx,g cm-3	1.177	1.263
Z	4	4
Mu (mm-1)	0.285	0.319
F000	3252.0	3484.0
F000'	3255.51	
h,k,lmax	21,26,28	17,20,21
Nref	16632	7805
Tmin,Tmax	0.855,0.920	0.844,0.986
Tmin'	0.855	

Correction method= # Reported T Limits: Tmin=0.844 Tmax=0.986
AbsCorr = MULTI-SCAN

Data completeness= 0.469   Theta(max)= 25.775

```
R(reflections)= 0.0898( 6081)          wR2(reflections)= 0.2301( 7805)

S = 1.096                   Npar= 958
```

The following ALERTS were generated. Each ALERT has the format
test-name_ALERT_alert-type_alert-level.
Click on the hyperlinks for more details of the test.

Alert level B

```
PLAT920_ALERT_1_B Theta(Max)  in CIF and FCF Differ by ..........       6.00 Degree
PLAT990_ALERT_1_B Deprecated .res/.hkl Input Style SQUEEZE Job ...          ! Note
```

Author Response: The first Alert_B is probably linked to an environment SHELXL problem. Therefore, this is not a serious problem, which have no impact in the validity of our model.

Alert level C

```
DIFMX02_ALERT_1_C  The maximum difference density is > 0.1*ZMAX*0.75
            The relevant atom site should be identified.
PLAT088_ALERT_3_C Poor Data / Parameter Ratio ..................       8.15 Note
PLAT094_ALERT_2_C Ratio of Maximum / Minimum Residual Density ....       2.41 Report
PLAT097_ALERT_2_C Large Reported Max.  (Positive) Residual Density       1.96 eA-3
PLAT213_ALERT_2_C Atom C54             has ADP max/min Ratio .....        3.4 prolat
PLAT220_ALERT_2_C NonSolvent   Resd 1  C   Ueq(max)/Ueq(min) Range        5.0 Ratio
PLAT222_ALERT_3_C NonSolvent Resd 1  H   Uiso(max)/Uiso(min) Range        4.7 Ratio
PLAT234_ALERT_4_C Large Hirshfeld Difference N6       --C38      .       0.17 Ang.
PLAT234_ALERT_4_C Large Hirshfeld Difference N8       --C59      .       0.18 Ang.
PLAT234_ALERT_4_C Large Hirshfeld Difference C28      --C29A     .       0.18 Ang.
PLAT234_ALERT_4_C Large Hirshfeld Difference C28      --C31A     .       0.24 Ang.
PLAT234_ALERT_4_C Large Hirshfeld Difference C28      --C31B     .       0.21 Ang.
PLAT234_ALERT_4_C Large Hirshfeld Difference C38      --C39      .       0.20 Ang.
PLAT234_ALERT_4_C Large Hirshfeld Difference N10      --C83      .       0.16 Ang.
PLAT242_ALERT_2_C Low    'MainMol' Ueq as Compared to Neighbors of         Fe Check
PLAT242_ALERT_2_C Low    'MainMol' Ueq as Compared to Neighbors of        C28 Check
PLAT242_ALERT_2_C Low    'MainMol' Ueq as Compared to Neighbors of        C38 Check
PLAT242_ALERT_2_C Low    'MainMol' Ueq as Compared to Neighbors of        C61 Check
PLAT341_ALERT_3_C Low Bond Precision on  C-C Bonds ..............    0.01334 Ang.
PLAT906_ALERT_3_C Large K Value in the Analysis of Variance ......      9.848 Check
PLAT906_ALERT_3_C Large K Value in the Analysis of Variance ......      2.258 Check
PLAT911_ALERT_3_C Missing FCF Refl Between Thmin & STh/L=    0.476         26 Report
                 2  0  0,   0  2  0,   1  2  0,   1  1  1,   0  2  1,   6 19  1,
                 0  0  2,  -1  1  2,   0  1  2,  -3  5  2, -16  7  3,  12 11  3,
                -8 18  3, -16  7  5,  -5 19  6,  -4 19  7, -16  4 10, -16  2 11,
                 6 15 11,  12  3 12,  -3 17 12, -11  9 16, -13  0 17, -11  0 19,
                -2 10 19,   0  4 21,
PLAT934_ALERT_3_C Number of (Iobs-Icalc)/Sigma(W) > 10 Outliers ..          1 Check
                -1  2  2,
PLAT971_ALERT_2_C Check Calcd Resid. Dens.  0.18Ang From C65            2.06 eA-3
PLAT971_ALERT_2_C Check Calcd Resid. Dens.  2.14Ang From C29B           1.83 eA-3
```

Alert level G

```
FORMU01_ALERT_2_G  There is a discrepancy between the atom counts in the
            _chemical_formula_sum and the formula from the _atom_site* data.
            Atom count from _chemical_formula_sum:C89 H101 Cl1 Fe1 K1 N10 O13
            Atom count from the _atom_site data:  C83 H96 Fe1 K1 N10 O13
CELLZ01_ALERT_1_G Difference between formula and atom_site contents detected.
CELLZ01_ALERT_1_G ALERT: Large difference may be due to a
            symmetry error - see SYMMG tests
           From the CIF: _cell_formula_units_Z    4
           From the CIF: _chemical_formula_sum  C89 H101 Cl Fe K N10 O13
           TEST: Compare cell contents of formula and atom_site data

           atom    Z*formula  cif sites diff
           C         356.00    332.00   24.00
           H         404.00    384.00   20.00
           Cl          4.00      0.00    4.00
           Fe          4.00      4.00    0.00
           K           4.00      4.00    0.00
           N          40.00     40.00    0.00
           O          52.00     52.00    0.00
PLAT002_ALERT_2_G Number of Distance or Angle Restraints on AtSite         16 Note
PLAT003_ALERT_2_G Number of Uiso or U(i,j) Restrained non-H Atoms           4 Report
PLAT019_ALERT_1_G _diffrn_measured_fraction_theta_full/*_max < 1.0      0.499 Report
PLAT041_ALERT_1_G Calc. and Reported SumFormula    Strings  Differ     Please Check
              Calc: C83 H96 Fe K N10 O13
              Rep.: C89 H101 Cl Fe K N10 O13
PLAT051_ALERT_1_G Mu(calc) and Mu(CIF) Ratio Differs from 1.0 by .      10.77 %
PLAT083_ALERT_2_G SHELXL Second Parameter in WGHT  Unusually Large      49.28 Why ?
PLAT171_ALERT_4_G The CIF-Embedded .res File Contains EADP Records          3 Report
PLAT172_ALERT_4_G The CIF-Embedded .res File Contains DFIX Records         13 Report
PLAT176_ALERT_4_G The CIF-Embedded .res File Contains SADI Records          1 Report
PLAT178_ALERT_4_G The CIF-Embedded .res File Contains SIMU Records          1 Report
PLAT186_ALERT_4_G The CIF-Embedded .res File Contains ISOR Records          1 Report
PLAT188_ALERT_3_G A Non-default SIMU Restraint Value has been used     0.0100 Report
PLAT199_ALERT_1_G Reported _cell_measurement_temperature ..... (K)        293 Check
PLAT200_ALERT_1_G Reported   _diffrn_ambient_temperature ..... (K)        293 Check
PLAT232_ALERT_2_G Hirshfeld Test Diff (M-X)  Fe       --O6B      .        6.5 s.u.
PLAT232_ALERT_2_G Hirshfeld Test Diff (M-X)  Fe       --O5A      .       12.3 s.u.
PLAT232_ALERT_2_G Hirshfeld Test Diff (M-X)  Fe       --O6A      .        6.2 s.u.
PLAT301_ALERT_3_G Main Residue  Disorder ..............(Resd    1)         7% Note
PLAT432_ALERT_2_G Short Inter X...Y Contact  O1A      ..C80      .       3.02 Ang.
                                               2-x,-y,1-z  =      3_756 Check
PLAT606_ALERT_4_G Solvent Accessible VOID(S) in Structure ........          ! Info
PLAT779_ALERT_4_G Suspect or Irrelevant (Bond) Angle(s) in CIF ...      41.00 Deg.
              C65  -O6B  -O5B     1_555   1_555   1_555 ......     #  269 Check
PLAT860_ALERT_3_G Number of Least-Squares Restraints .............         58 Note
PLAT869_ALERT_4_G ALERTS Related to the Use of SQUEEZE  Suppressed          ! Info
PLAT883_ALERT_1_G No Info/Value for _atom_sites_solution_primary .     Please Do !
PLAT909_ALERT_3_G Percentage of I>2sig(I) Data at Theta(Max) Still        69% Note
PLAT910_ALERT_3_G Missing # of FCF Reflection(s) Below Theta(Min).          4 Note
                1  1  0,  -1  0  1,   1  0  1,   0  1  1,
PLAT933_ALERT_2_G Number of HKL-OMIT Records in Embedded .res File         14 Note
               -1  0  1,   1  1  0,   0  1  1,   0  0  2,   1  0  1,  -1  1  2,
                1  1  1,   2  0  0,   0  1  2,   0  2  0,   1  2  0,   0  2  1,
               -3  5  2,  12 11  3,
PLAT950_ALERT_5_G Calculated (ThMax) and CIF-Reported Hmax Differ           4 Units
PLAT951_ALERT_5_G Calculated (ThMax) and CIF-Reported Kmax Differ           6 Units
```

```
PLAT952_ALERT_5_G Calculated (ThMax) and CIF-Reported Lmax Differ.          7 Units
PLAT956_ALERT_1_G Calculated (ThMax) and Actual (FCF) Hmax Differ           4 Units
PLAT957_ALERT_1_G Calculated (ThMax) and Actual (FCF) Kmax Differ           6 Units
PLAT958_ALERT_1_G Calculated (ThMax) and Actual (FCF) Lmax Differ.          7 Units
PLAT961_ALERT_5_G Dataset Contains no Negative Intensities .......     Please Check
PLAT965_ALERT_2_G The SHELXL WEIGHT Optimisation has not Converged     Please Check
PLAT969_ALERT_5_G The 'Henn et al.' R-Factor-gap value ..........      5.354 Note
              Predicted wR2: Based on SigI**2  4.30 or SHELX Weight 21.00
PLAT978_ALERT_2_G Number C-C Bonds with Positive Residual Density.          1 Info
```

```
  0 ALERT level A = Most likely a serious problem - resolve or explain
  2 ALERT level B = A potentially serious problem, consider carefully
 25 ALERT level C = Check. Ensure it is not caused by an omission or oversight
 40 ALERT level G = General information/check it is not something unexpected

 14 ALERT type 1 CIF construction/syntax error, inconsistent or missing data
 21 ALERT type 2 Indicator that the structure model may be wrong or deficient
 12 ALERT type 3 Indicator that the structure quality may be low
 15 ALERT type 4 Improvement, methodology, query or suggestion
  5 ALERT type 5 Informative message, check
```

It is advisable to attempt to resolve as many as possible of the alerts in all categories. Often the minor alerts point to easily fixed oversights, errors and omissions in your CIF or refinement strategy, so attention to these fine details can be worthwhile. In order to resolve some of the more serious problems it may be necessary to carry out additional measurements or structure refinements. However, the purpose of your study may justify the reported deviations and the more serious of these should normally be commented upon in the discussion or experimental section of a paper or in the "special_details" fields of the CIF. checkCIF was carefully designed to identify outliers and unusual parameters, but every test has its limitations and alerts that are not important in a particular case may appear. Conversely, the absence of alerts does not guarantee there are no aspects of the results needing attention. It is up to the individual to critically assess their own results and, if necessary, seek expert advice.

Publication of your CIF in IUCr journals

A basic structural check has been run on your CIF. These basic checks will be run on all CIFs submitted for publication in IUCr journals (*Acta Crystallographica*, *Journal of Applied Crystallography*, *Journal of Synchrotron Radiation*); however, if you intend to submit to *Acta Crystallographica Section C* or *E* or *IUCrData*, you should make sure that full publication checks are run on the final version of your CIF prior to submission.

Publication of your CIF in other journals

Please refer to the *Notes for Authors* of the relevant journal for any special instructions relating to CIF submission.

PLATON version of 13/05/2024; check.def file version of 04/05/2024

Datablock I - ellipsoid plot

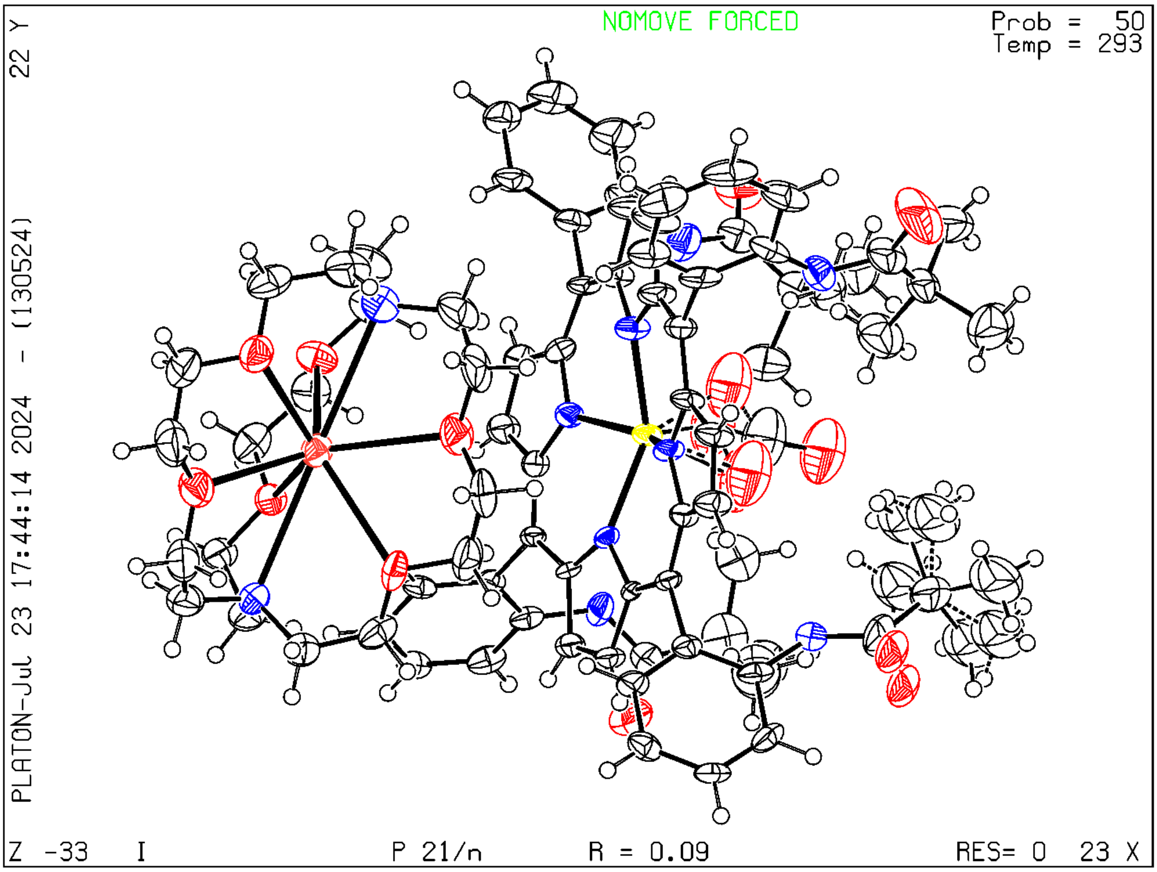

Supplement: Supplementary file 1 [file molecules-29-03722-s001.zip › C-New Checkcif-Fe3TpivPP(CO3-23-07-2024.pdf]
