# Supplementary material for: Synthesis, X-ray Crystallography, Spectroscopic Characterizations, Density Functional Theory, and Hirshfeld Surface Analyses of a Novel (Carbonato) Picket Fence Iron(III) Complex
Source: Molecules. 2024 Aug 6;29(16):3722. doi: 10.3390/molecules29163722 (PMC11357499; doi:10.3390/molecules29163722)
Supplement: Supplementary file 1 [file molecules-29-03722-s001.zip › Supplementary Materials.pdf]

Synthesis, X-ray Crystallography, Spectroscopic Characterizations, Density Functional Theory, and Hirshfeld Surface Analyses of a Novel (carbonato) picket fence iron(III) complex

Mondher Dhifet[a,b], Bouzid Gassoumi[c], Maxim Lutoshkin[d] , Anna S Kazachenko[e], S. KAZACHENKO[f] Omar M. Al-Dossary[g], Noureddine ISSAOUI[h*] and Habib Nasri[a]

[a] University of Monastir, Laboratory of Physical Chemistry of Materials (LR01ES19), Faculty of Sciences of Monastir, Avenue of the Environment, 5019 Monastir, Tunisia.
[b] University of Gafsa, Faculty of Sciences of Gafsa.
[c] Laboratory of Advanced Materials and Interfaces (LIMA), University of Monastir, Faculty of Science of Monastir, Avenue of Environnment, 5000 Monastir, Tunisia.
[d] Institute of Chemistry and Chemical Technology SB RAS, FRC KSC SB RAS,Russian Federation, 660036, Krasnoyarsk, Akademgorodokst., 50/24
[e] Siberian Federal University, Russian Federation, 660041, Russian Federation, Krasnoyarsk, pr. Svobodny, 79
[f] Institute of Chemistry and Chemical Technology SB RAS, Federal Research Center, Krasnoyarsk Science Center SB RAS", Akademgorodok, 50/24, Krasnoyarsk, 660036, Russia
[g] Department of Physics and Astronomy, College of Science, King Saud University, PO Box 2455, Riyadh 11451, Saudi Arabia.
[h] Laboratory of Quantum and Statistical Physics LR18ES18, Faculty of Sciences of Monastir, Avenue of the Environment, Monastir, 5079, Tunisia.
[*] E-mail: issaoui_noureddine@yahoo.fr

Contents

1. Synthesis of the picket fence porphyrin (**H_2TpivPP**)……………………………………………….................**2**

2. UV/Vis spectroscopy……………………………………………………………………..…**3**

3. X-ray molecular structure of complex **I**……………………………..…………....….....................**4**

1. Synthesis of the picket fence porphyrin (H_2TpivPP)

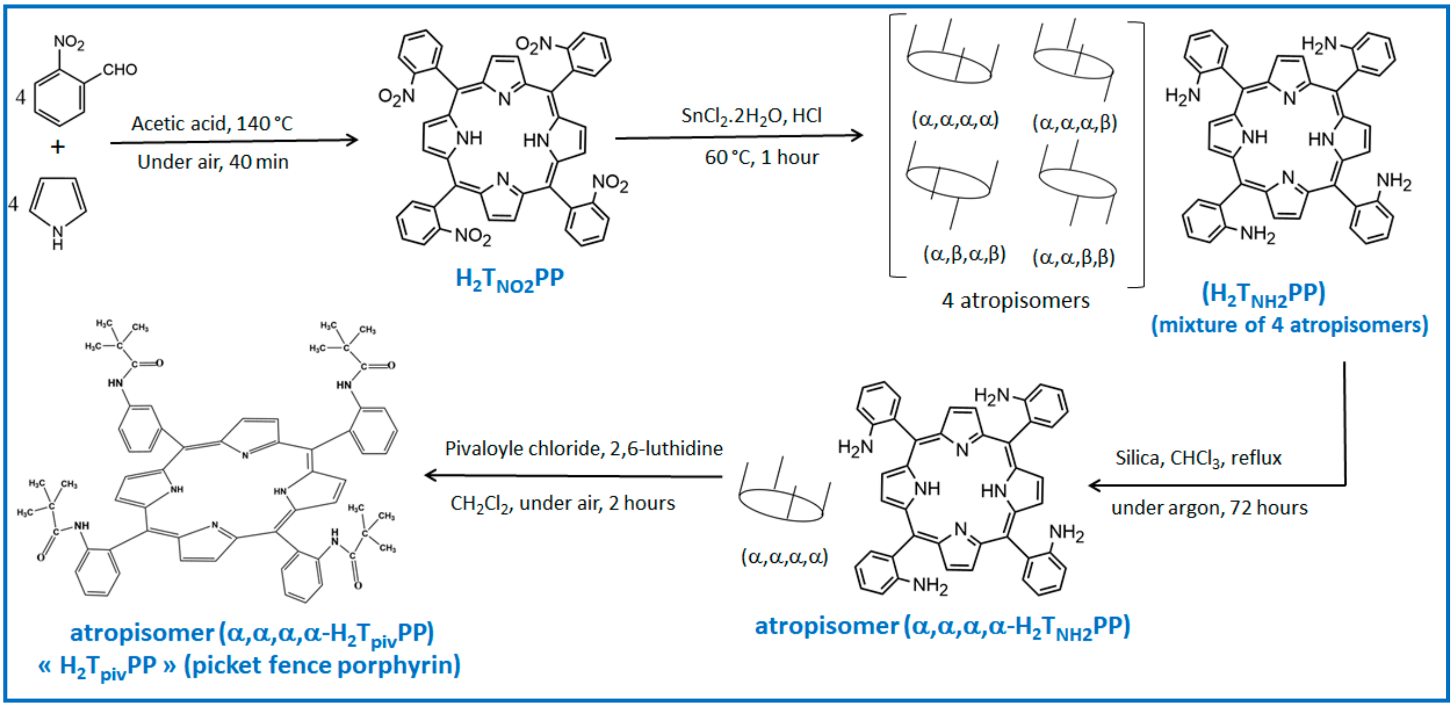

Scheme S1. Scheme of the preparation of the picket fence porphyrin (H_2TpivPP).

2. UV/Vis spectroscopy

Table S1. Electronic absorption data for complex **I** and a selection of *meso*-arylporphyrin compounds.

**Complex**	**λmax (nm)**					**Solvents**[e]	**Ref.**
	**Soret region**	**β, α region**					
Meso-arylporphyrins							
$H_2TpivPP$	**416**	514	548	590	646	CH_2Cl_2	t.w.
H_2TPP [a]	**418**	515	549	591	647	CH_2Cl_2	[1]
H_2TBrPP [b]	**419**	515	549	590	648	CH_2Cl_2	[2]
H_2TPBP [c]	**419**	514	551	590	646	CH_2Cl_2	[3]
H_2TClPP [d]	**418**	515	550	549	647	CH_2Cl_2	[4]
Iron(III) porphyrins							
$[Fe^{III}(TpivPP)Cl]$	**417**		506	575	603	C_6H_5Cl	t.w.
$[Fe^{III}(TpivPP)(CO_3)]^-$	**424**		510	567	607	C_6H_5Cl	t.w.
$[Fe^{III}(TpivPP)(OAc)]$	**414**		505	574	-	C_6H_5Cl	[5]
$[Fe^{III}(TpivPP)(NCS)]$	**417**		513	586	-	C_6H_5Cl	[6]
$[Fe^{III}(TpivPP)(NCO)]$	**414**		508	576	-	C_6H_5Cl	[7]
$[Fe^{III}(TpivPP)(NO_2)_2]^-$	**426**		553	-	-	C_6H_5Cl	[8]
$[Fe^{III}(TpivPP)(NO_2)(py)]$	**420**		546	-	-	C_6H_5Cl	[9]

[a] H_2TPP = *meso* –tetraphenylporphyrin; [b] H_2TBrPP = 4-bromophenylporphyrin, [c] H_2TPBP = 4-(benzoyloxy)phenyl] porphinato; [d] H_2TClPP = meso-tetrakis(4-chlorophenyl)porphyrin; ; [e]: C_6H_5Cl is chlorobenzene and CH_2Cl_2 is dichlorometane ; t.w: this work.

[1] K. Ezzayani, A. Ben Khelifa, E. Saint-Aman, F. Loiseau, H. Nasri, Synthesis, spectroscopic characterizations, cyclic voltammetry investigation and molecular structure of the di-μ-cyanato-*N*-bis(μ-1,4,7,10,13,16-hexaoxacyclooctadecane)bis(5,10,15,20-tetraphenylporphyrinato) dimagnesiumdipotassium complex, Polyhedron 117 (2016) 817, doi.org/10.1016/j.poly.2016.06.045.

[2] N. Amiri, F.B. Taheur, S. Chevreux, E. Wenger, G. Lemercier, H. Nasri, Synthesis, crystal structure and spectroscopic characterizations of porphyrin-based Mg (II) complexes–Potential application as antibacterial agent, Tetrahedron 73 (2017) 7011, doi.org/10.1016/j.tet.2017.10.029.

[3] N. Amiri, M. Hajji, F.B. Taheur, S. Chevreux, T. Roisnel, G. Lemercier, H. Nasri, Two novel magnesium (II) meso-tetraphenylporphyrin-based coordination complexes: Syntheses, combined experimental and theoretical structures elucidation,spectroscopy, photophysical properties and antibacterial activity, J. Solid State Chem. 258 (2018) 477, doi.org/10.1016/j.jssc.2017.11.018.

[4] T. Fradi, O. Noureddine, F.B. Taheur et al., New DMAP meso-arylporphyrin Magnesium(II) complex. Spectroscopic, Cyclic voltammetry and X-ray molecular structure characterization. DFT, DOS and MEP calculations and Antioxidant and Antifungal activities,Journal of Molecular Structure1236 (2021) 130299, doi.org/10.1016/j.molstruc.2021.130299.

[5] E. L. Bominaar, X. Q. Ding, A. Gismelseed, E. Bill, H. Winkler, A. X. Trautwein, H. Nasri, J. Fischer, R. Weiss, Structural, Moessbauer, and EPR investigations on two oxidation states of a five-coordinate, high-spin synthetic heme. Quantitative interpretation of zero-field parameters and large quadrupole splitting, Inorg. Chem., 31 (1992) 1845, doi.org/10.1021/ic00036a023.

[6] H. Nasri, M. Debbabi, Synthesis, spectroscopic and structural characterization of the pentacoordinate high-spin Fe (III) isothiocyanate "picket fence" porphyrin complex, Polyhedron 17 (1998) 3607, doi.org/10.1016/S0277-5387(98)00156-9.

[7] M.S. Belkhiria, M. Dhifet, H. Nasri, Preparation and spectroscopic properties of the (cyanato-N) and (oxalato) iron(III) "picket fence" porphyrins: Structure of the (cyanato-N)(α,α,α,α-tetrakis(o-pivalamidophenyl)porphinato)iron(III) complex, J. Porphyrins and Phthalocyanines, 9 (2005) 575, doi.org/10.1142/S108842460500068X.

[8] H. Nasri, J. A. Goodwin, W.R. Scheidt, Use of protected binding sites for nitrite binding in iron(III) porphyrinates. Crystal structure of the bis(nitro)(.alpha.,.alpha.,.alpha.,.alpha.-tetrakis(o-pivalamidophenyl)porphinato)iron(III) anion, Inorg. Chem., 28 (1990) 185, doi.org/10.1021/ic00327a009.

[9] H. Nasri, Y. Wang, B.H. Huynh, F.A. Walker, W.R. Scheidt, Reactions of bis(nitro)(.alpha.,.alpha.,.alpha.,.alpha.-tetrakis(o-pivalamidophenyl)porphinato)ferrate(III) with pyridine and imidazole. EPR and Moessbauer spectra and molecular structures of the mixed-ligand species, Inorg. Chem. 30 (1991) 1483, doi.org/10.1021/ic00007a012.

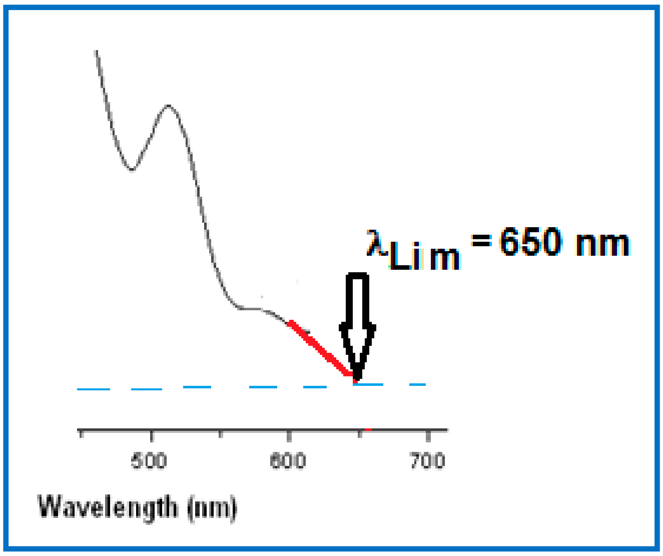

Fig. S1. Region of Q bands of complex **I**. Determination of the λ_{Lim}.

3. X-ray molecular structure of complex I

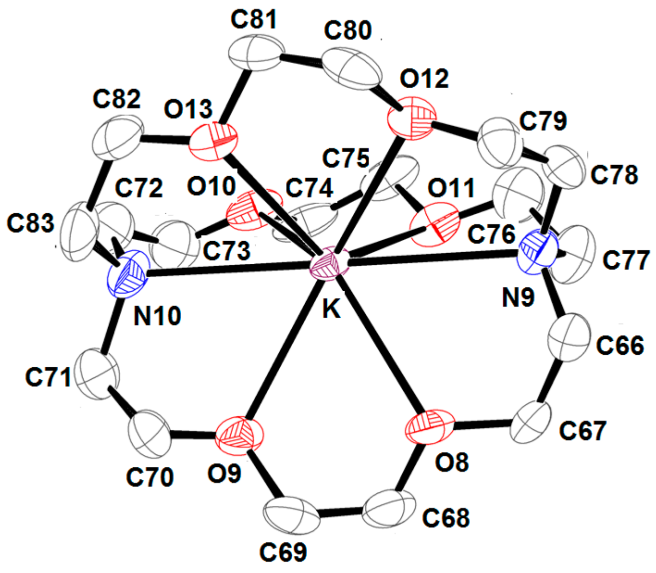

Fig. S2. ORTEP view of [K(crypt-222)]$^+$ counterion, the thermal ellipsoids are drawn at the 30% probability level. Hydrogen atoms have been omitted for clarity.
